# Enhanced Insulin Production From Porcine Islets: More Insulin, Less Islets

**DOI:** 10.3389/ti.2024.13954

**Published:** 2024-12-18

**Authors:** Nizar I. Mourad, Pierre Gianello

**Affiliations:** Pôle de Chirurgie Expérimentale et Transplantation, Université Catholique de Louvain, Brussels, Belgium

**Keywords:** porcine islets, insulin secretion, transplantation, genetic modification, diabetes

## Abstract

Clinical pancreatic islet xenotransplantation will most probably rely on genetically modified pigs as donors. Several lines of transgenic pigs carrying one and more often, multiple modifications already exist. The vast majority of these modifications aim to mitigate the host immune response by suppressing major xeno-antigens, or expressing immunomodulatory molecules that act locally at the graft site. While these modifications are essential and have proven beneficial in preclinical trials, ensuring good intrinsic islet secretory function is equally important to achieve normoglycemia in recipients. Neonatal and even adult porcine islets are known for their low secretory response to physiological stimulation, a shortcoming that is often overcome by implanting extremely large numbers of such islets to compensate for insulin requirement incompatibilities between donor pigs and rodent, non-human primate or human recipients. Recent studies have revealed the existence of secretory amplifying pathways in porcine beta-cells previously identified in murine and human cells. Building upon these findings, a new line of transgenic pigs where these pathways are activated specifically in beta-cells has been created. Compared to their wild-type counterparts, islets from these transgenic pigs have proven to be better insulin secretors in their native pancreas environment, *in vitro* after isolation and most importantly *in vivo* after transplantation to diabetic mice.

## Introduction

Islet replacement therapies aiming to restore insulin independency in type I diabetic patients need to ensure that transplanted cells from human or animal origin produce enough insulin to sustain the host’s physiological needs and establish efficient glucose homeostasis. This is also true even when the therapy’s ambitions are limited to improving the patient’s quality of life by diminishing exogenous insulin doses or alleviating the risk of severe hypoglycemia as seen in difficult-to-manage brittle diabetes. Surgical and immunological considerations such as the host immune response, implantation site, graft vascularization and its access to nutrients and oxygen as well as its capacity to deliver the secreted hormones all weight in the successful outcome of the procedure but intrinsic secretory properties of the grafted cells remain an undeniably determinant factor. From a qualitative point of view, porcine insulin is almost identical to its human counterpart differing only by one amino acid at position 30 in the B-chain (alanine in pigs vs. threonine in humans) [1]. Quantitatively, insulin secretion from porcine islets is significantly low compared to secretion from human islets in response to the same stimuli [2]. While everybody agrees on this specificity of porcine islets, different strategies have been proposed to overcome this limitation with most research groups resorting to increasing the number of implanted cells to compensate low secretion, promoting cell differentiation and maturation *in vitro* especially when using neonatal islets or using genetic modifications to improve the secretory capacity of porcine islets as we have recently suggested. In the present review, we briefly summarized our current knowledge of porcine islet physiology, their efficacy in experimental xenotransplantation models with particular emphasis on the development of genetically modified, functionally enhanced islets.

## Glucose Homeostasis and Insulin Requirements in Pigs, Humans and Non-Human Primates

In the context of xenotransplantation, immune incompatibilities seem like the most obvious barrier hindering graft survival and its subsequent function. Beyond this, functional physiological compatibility between donor and recipient species is equally decisive. It is therefore worthy looking at differences and similarities of glucose regulation in species of interest. During an intravenous glucose tolerance test (IVGTT), the quick rise in glycaemia is paralleled by a rapid sevenfold increase of insulinemia in macaques compared to a much slower lingering rise in pigs [3]. Moreover, fasting glucose was found to be lower whereas insulin and c-peptide were significantly higher in non-human primates compared to pigs [4]. In humans, the situation is intermediate although somehow closer to that of pigs regarding glycaemia and c-peptide values [4]. Interestingly, in pigs, the impact of glucose changes on glucagon secretion seems greater and faster to occur than on insulin secretion [5]. These discrepancies suggest a relative physiological incompatibility between islets from porcine and human origin and even more so with non-human primates. Quantitative and/or qualitative adaptations of porcine islets seem then necessary to compensate for this shortcoming.

## What Do in Vitro Studies Tell US?

**FIGURE 1:**
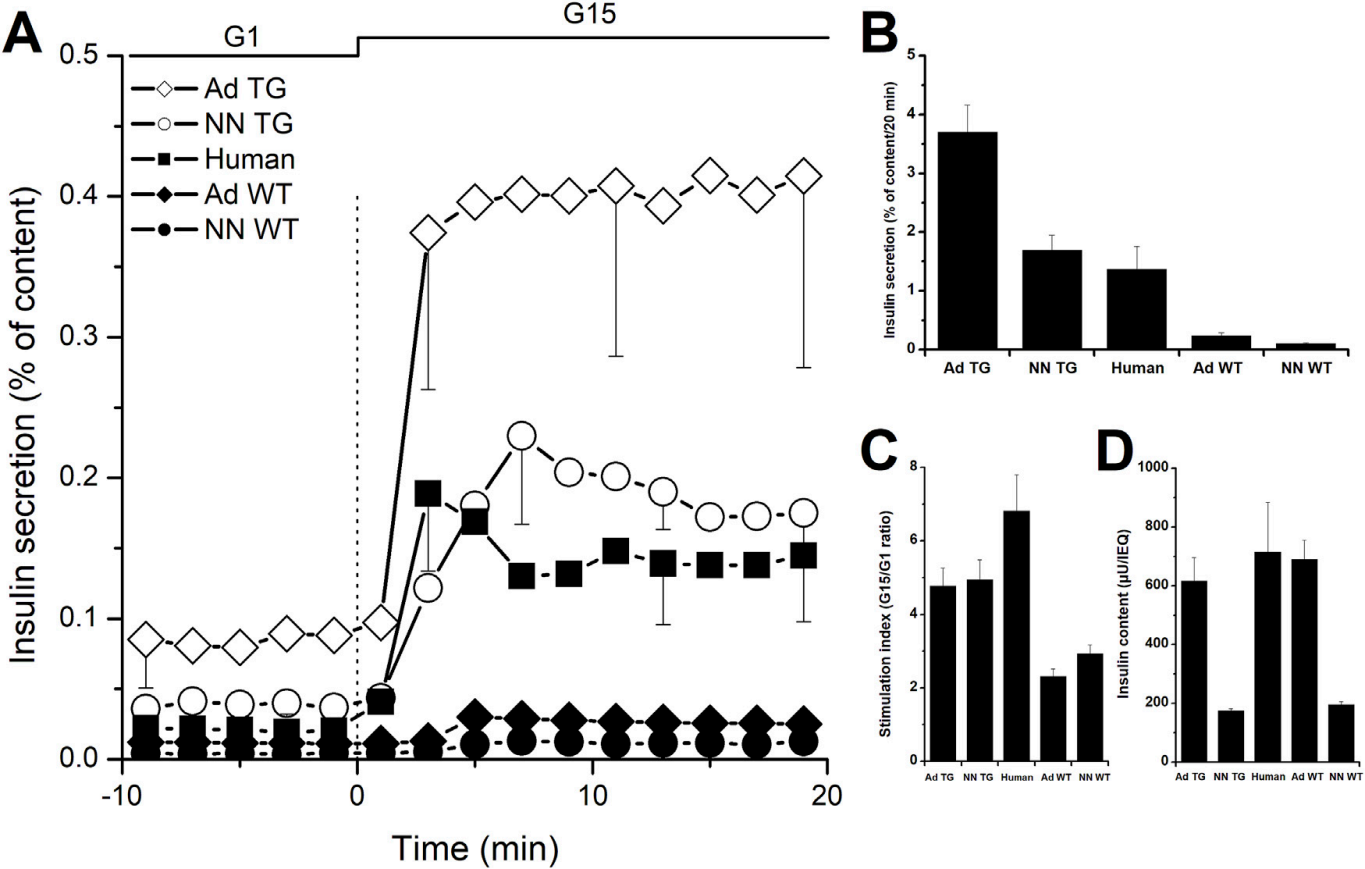
*In vitro* insulin secretion from wild-type and InsGLP-1M3R isolated islets. Dynamic islet perifusion experiments were performed to evaluate islet secretory response. **(A)**: batches of 1000 IEQ islets from neonatal (NN) wild-type (WT, closed symbols) and InsGLP-1M3R (TG, open symbols) piglets or 200 IEQ islets from adults (Ad) were perifused with 1 mM glucose (G1) then stimulated with 15 mM glucose (G15) as shown on top of the figures. Similar experiments performed using human islets (closed squares) are shown for comparison. **(B)**: Area under the curve integration of insulin secretion during 20 min of G15 stimulation is represented. **(C)**: Stimulation indices (G15/G1 ratio) are shown. In all experiments, islets in perifusion chambers were retrieved at the end of the experiments and lysed to determine total insulin content shown in **(D)**. Results are expressed as percentages of total islet insulin content. Values are means ± SE from n = 10–50 experiments.

Glucose-stimulated insulin secretion from isolated islets can be thoroughly studied *in vitro* using static incubation and/or dynamic perifusion assays therefore providing insight into the function of these islets that can be predictive of their capacity to alleviate or heal diabetes once transplanted. Ideally, grafted islets should secrete the same amount of insulin as the host’s native islets when exposed to similar stimuli. However, we know that porcine islets secrete down to 10-times less insulin compared to human or non-human primates islets when challenged with 15 mM glucose as evidenced by a lower stimulation index (the ratio of stimulated to unstimulated secretion; Figure 1C) and a much smaller area under the curve (AUC) for insulin secretion curves (Figure 1B) [6–10]. Unsurprisingly, the difference between porcine and human islets becomes even more striking when using islets from neonatal piglets as their immature cells contain less insulin and therefore secrete less of it as previously shown in our own [6] and other studies [9, 11–15]. Interestingly, such islets present the advantage of undergoing further maturation *in vitro* especially if cultured under optimized conditions, receiving growth and maturation factors from a specific culture medium and physical and structural support from encapsulation on a collagen matrix [16].

From a qualitative point of view, the secretory response of porcine islets to glucose stimulation is characterized by a relatively small first phase, an ascending second phase (Figure 1A) as well as a paradoxical off-response i.e., an unexpected short-lived increase of insulin secretion upon return to a resting glucose concentration [17]. Some have attributed this to the small number of alpha-cells in porcine islets (8% vs. 30% in porcine and human islets respectively) [18], the majority of which is further lost during isolation due to their peripheral localization and the lack of a peri-insular capsule [19]. However, this hypothesis has been challenged by more recent findings showing similar composition and distribution of alpha-cells in human and porcine islets [20]. Nonetheless, glucagon has been shown to amplify insulin secretion from porcine islets [21]. However, this cannot explain low insulin secretion from native porcine islets *in vivo* as previously mentioned.

A few studies attempted to investigate mechanisms that can potentially explain the weak secretory response of pig islets. However, no defects were found in glucose sensing, transport, phosphorylation and utilization which were all comparable to what is observed in rodent and human islets [22, 23]. On the other hand, we [2] and others [24] have observed that glucose-induced cytosolic calcium changes in porcine beta-cells are small and delayed compared to rodent [25] and human [26] islets and that typical glucose-induced calcium oscillations, a hallmark of stimulus-secretion coupling in pancreatic beta-cells, were not observed in individual porcine islets [2].

Glucose also modulates the amplitude of the insulin secretory response via a non-calcium-dependent mechanism known as metabolic amplification [27]. This mechanism first investigated in rodents and later demonstrated in human islets [28] has been shown to be equally operational in porcine islets as demonstrated by experiments using ATP-dependent potassium channels (KATP) modulators [7]. In most of the aforementioned experiments, exposure of porcine islets to adenylyl cyclase activators or other pharmacological substances inducing an increase of cAMP concentrations always resulted in a significantly increased secretory response to glucose stimulation, a principle that we recently utilized to produce modified functionally enhanced porcine islets as discussed later in this review.

## Pig Islet Xenotransplantation

In the research setup, two main models have been used to evaluate efficacy of porcine islets in treating or alleviating chemically- or surgically-induced type-I diabetes: pig-to-non-human primate and pig-to-rodent. While both models have their pros and cons, they both represent a particularly challenging environment for porcine islets. As discussed earlier, non-human primates have higher insulin requirements than both pigs and humans [4] while rodent insulin differs substantially from its porcine counterpart [29] lessening efficacy of the latter in mice and rats. Therefore, one could expect porcine islets to underperform in both cases. This is particularly exemplified by the extremely large number of islets that are necessary to exert an effect in such experiments. Nonetheless, several studies have reported successful long-term reversal of diabetes following transplantation of free [30–37] or encapsulated [38–40] porcine islets in preclinical non-human primate models.

As indicated in Table 1, transplanted islet dose ranged from 10,000 to 100,000 IEQ/kg recipient body weight when using adult pigs as islet donors and 9,000 to 115,000 when using islets from neonatal piglets. Free islets are usually injected in the portal vein, thus mimicking clinical practice in human islet allotransplantation but exposing islets to IBMIR known to cause substantial early islet mass loss and impaired secretory function due to mitochondrial damage in surviving islets [41]. On the other hand, encapsulated islets are transplanted subcutaneously or in the peritoneal space where the main limitation is oxygenation [39] and access to nutrients during the critical period between implantation and graft vascularization.

**TABLE 1:** Experimental pig-to-NHP and pig-to-rodent islet xenotransplantation.

Recipient	Donor age	Site	Islet dose (IEQ/kg)	1st author	Year	Ref
Cynomolgus	Adult	SC	20,000	Ludwig	2017	37
Cynomolgus	Adult	P	48,000–91 000	Safley	2018	38
Rhesus	Adult	IP	77,000–100 000	Shin	2018	30
Rhesus	Adult	IP	87,000–100 000	Min	2018	31
Rhesus	NN	IP	39,000–115 000	Gao	2021	32
Rhesus	Adult	IP	55,000–100 000	Kim	2021	33
Cynomolgus	Adult	IP	20,130	Graham	2022	35
Baboons	NN	IP	9,000–56 000	Hawthorne	2022	36
Cynomolgus	Adult	P	9,800–22 900	Holdcraft	2022	39
Mice	NN	P	80,000	Kobayashi	2006	47
Mice	NN	KC/IM	100,000–120 000	Wolf-van Buerck	2015	44
Mice	Adult	SC	20,000	Yu	2020	43
Mice	Adult	P	400,000	Ajima	2023	46
Mice	NN	KC/SC	32,000–160 000	Citro	2023	42
Mice	NN	KC	100,000	Mourad	2024	45

Non-human primates (NHP) and mice received pancreatic islets from adult or neonatal (NN) pigs. Transplanted islet dose is indicated as IEQ/kg recipient body weight. For mice experiments, IEQ/kg was calculated assuming a body weight of 25 g/mouse. Islets were transplanted in the portal vein (IP), subcutaneously (SC), in the peritoneal space (P), under the kidney capsule (KC) or in an intramuscular (IM) site.

Immunosuppression whether administered systemically to the recipient and/or created locally by means of genetic modification to the donor cells can help alleviate IBMIR and other immune rejection mechanisms [42] therefore improving transplantation outcome and even decreasing the number of islets required for efficient diabetes reversal [37]. In the case of encapsulated islets, oxygen supplementation either directly to the encapsulation device [38] or indirectly by pre-vascularizing the graft site or including angiogenic factors in encapsulation gels can help minimize islet loss due to hypoxia.

The pig-to-rodent model is easier to implement due to obvious cost-related as well as regulatory and ethical considerations and has been extensively used to refine experimental procedures before moving on to large animal models. Another advantage of mice models is the availability of immunodeficient mice strains permitting xenotransplantation studies without possible interference from immunosuppressant treatments. As such, these studies have focused more on finding the optimal implantation site or engineering it [43, 44]. The kidney subcapsular space is often used as a golden standard implantation site in these experiments using free islets [43, 45, 46] whereas encapsulated islets are typically implanted in the peritoneal [47, 48] or subcutaneous [44] space.

As shown in Table 1, mice received doses of up to 400,000 IEQ/kg [47], the minimal reported dose being 20,000 IEQ/kg [44]. In this context, an interesting approach was to adapt the transplanted islet dose to the daily insulin requirements of diabetic rat recipients. Agarose-encapsulated neonatal porcine islets were incubated *in vitro* to determine their daily insulin output and the number of macrocapsules to be implanted was adjusted to equal the amount of insulin units necessary to maintain normoglycemia [49].

## Genetic Modifications to Improve Porcine Islet Insulin Secretion

As mentioned earlier, most *in vitro* studies successfully used cAMP-increasing molecules to amplify glucose-induced insulin secretion from porcine islets [7] suggesting that insulin synthesis and content are not limiting factors. Indeed, intra-islet insulin content is similar in adult porcine and human islets but expectedly 3-times lower in neonatal piglet islets as shown in Figure 1D.

From numerous studies using murine islets and a growing number of studies using human islets, it has been established that hormonal and neural amplifying pathways exist in pancreatic beta-cells. Agents that bind GLP-1 receptors or directly activate adenylyl cyclase induce a rise in cAMP leading to activation of protein kinase-A (PKA) and Epac2, two protein kinases known to amplify glucose-stimulated insulin secretion mainly by increasing the number of readily releasable insulin granules. The parasympathic cholinergic neural amplifying pathway involves binding of acetylcholine to type-III muscarinic receptors (M3R), activation of membrane phospholipase leading to accumulation of diacylglycerol in the cytosol which activates protein kinase-C (PKC), another protein kinase involved in priming insulin granules to render them readily releasable. Using chemical activators of these pathways, we demonstrated that they exert an amplifying effect on porcine islet insulin secretion and even reported an unexpected synergetic effect when both pathways are activated in neonatal and adult porcine islets by means of pharmacological agents or adenoviral expression of a mutated GLP-1 and a constitutively activated form of M3R(6). In this model, substituting alanine at position 8 by a serine in the GLP-1 sequence prolongs the peptide half-life by rendering it resistant to dipeptidyl peptidase whereas a single-point mutation at position 490 in the M3R sequence renders the receptor constitutively activated [6]. Putting these two sequences under a porcine insulin promoter, we created an expression cassette (InsGLP-1M3R) that was then used to generate a line of transgenic pigs with beta-cell targeted expression [46].

As shown in Figure 1A, neonatal and adult islets isolated from these pigs secrete 16- to 15-times more insulin respectively compared to their wild-type counterparts. Compared to human islets, fold-increases are 1.2 and 2.7 for neonatal and adult InsGLP-1M3R islets respectively as illustrated in Figure 1B. In terms of stimulation index calculated as the ratio of secretion at high glucose to that at low glucose, InsGLP-1M3R islets were more responsive to glucose stimulation 4.9 vs. 2.9 for neonatal islets and 4.8 vs. 2.3 for adult islets. In comparison, we calculated an index of 6.8 for human islets (Figure 1C).

Recently, we reported using InsGLP-1M3R islets to treat streptozotocin-induced diabetes in immunodeficient mice [46]. These experiments show that 100% of InsGLP-1M3R islet recipients became normoglycemic following islet transplantation under the kidney capsule. In comparison, only 22% of wild-type islet recipients were successfully treated. Despite implanting the same amount of both islet types (2500 IEQ), we observed significantly higher porcine c-peptide values in the InsGLP-1M3R group (79 pM) compared to the wild-type group (34 pM; median values) [46]. These results strongly highlight the importance of secretory capacity of transplanted islets for a successful outcome. This is particularly relevant when using islets from neonatal piglets that are easier and cheaper to isolate besides being capable of undergoing further maturation and proliferation *in vivo* after transplantation [12] and even *in vitro* when cultured on a collagen matrix [16]. Interestingly, we did not observe any deterioration of transgenic islet function throughout the 9-month follow-up period in contrast to what has been reported in patients receiving sulfonylurea treatment to enhance their endogenous insulin production [50, 51]. Indeed, the InsGLP-1M3R modification acts by activating amplifying mechanisms without affecting the triggering pathway of insulin secretion whereas sulfonylureas directly bind K_ATP_ channels thus triggering insulin secretion. On the longer term, this is further supported by our observation that InsGLP-1M3R pigs maintain their glucose homeostasis and their improved insulin secretion from birth through adult age of up to 4 years. In conclusion, genetic modifications aiming to improve porcine islet insulin secretion such as the one discussed here can be as instrumental as immunology-oriented modifications in guaranteeing sufficient graft function to achieve normoglycemia.
